# Lattice light sheet imaging of membrane nanotubes between human breast cancer cells in culture and in brain metastases

**DOI:** 10.1038/s41598-017-11223-y

**Published:** 2017-09-08

**Authors:** Ian Parker, Katrina T. Evans, Kyle Ellefsen, Devon A. Lawson, Ian F. Smith

**Affiliations:** 1 Department of Neurobiology and Behavior, University of California, Irvine, CA USA; 2 Department of Physiology and Biophysics, University of California, Irvine, CA USA

## Abstract

Membrane nanotubes are cytosolic protrusions with diameters <1 µm that extend between cells separated by tens of µm. They mediate several forms of intercellular communication and are upregulated in diverse diseases. Difficulties in visualizing and studying nanotubes within intact tissues have, however, prompted skepticism regarding their *in vivo* relevance, and most studies have been confined to cell culture systems. Here, we introduce lattice-light sheet imaging of MDA-MB-231 human breast cancer cells genetically engineered to brightly express membrane–targeted GFP as a promising approach to visualize membrane nanotubes *in vitro* and *in situ*. We demonstrate that cultured cells form multiple nanotubes that mediate intercellular communication of Ca^2+^ signals and actively traffic GFP-tagged membrane vesicles along their length. Furthermore, we directly visualize nanotubes *in situ*, interconnecting breast cancer cells in live acute brain slices from an experimental mouse model of breast cancer brain metastasis. This amenable experimental system should facilitate the transition of the study of intercellular communication by membrane nanotubes from cell culture to the whole animal.

## Introduction

Cell-to-cell communication is vital for coordinating the development and proper functioning of tissues and organs. Cells have long been known to communicate using well-established mechanisms, such as through the secretion of soluble factors such as chemokines, and through membrane-associated proteins, extracellular vesicles, gap junction channels and synapses. Recently, a new form of cellular communication was proposed, whereby long and thin extensions of the plasma membrane relay specific and targeted messages between cells separated by tens of microns. These membrane projections have been given a diverse nomenclature, including tunneling membrane nanotubes, membrane tethers, tumor microtubes, cytonemes, airynemes and specialized signaling filopodia^1–6^; names defined by morphological and functional profiling such as cytoskeletal composition, size, types of cargo they are able to transmit and the experimental system in which they are observed. Here we use the generic term nanotubes for these structures, which possess the intriguing potential to establish direct, long distance ‘wired’ connections enabling greater specificity than communication via the secretion of extracellular soluble messengers, such as in endocrine, paracrine or exosome signaling.

Cell-to-cell communication between cancer cells within the tumor microenvironment has been shown to play a vital role in disease progression and resistance to therapies. In this context a number of recent studies have demonstrated the increasing role for membrane protrusions variously termed tumor microtubes^5, 7, 8^ and nanoscale bridges^9^ in the pathogenesis and spread of tumors. However, the lack of a specific marker and imaging methodologies to visualize nanotubes has hindered their morphological and physiological studies within intact live tissues. Although several reports document microtube-like structures in resected surgical sections from human lung^10, 11^, ovarian^12^ and laryngeal cancers^13^, these preparations are not readily amenable to *in situ* study and manipulation.

Here we introduce the use of lattice-light sheet microscopy (LLSM) to image membrane nanotubes *in vitro* and *in situ*. This form of microscopy uses a linear array of coherent Bessel-Gaussian beams to create an extremely thin sheet of light, that is stepped through the specimen to rapidly obtain unprecedented spatiotemporal resolution in three-dimensions over time^14^. Importantly this approach minimizes photo-toxicity and photobleaching as compared to conventional imaging approaches, thereby facilitating viable long-term imaging. To visualize membrane nanotubes we employed a human breast cancer cell line^15^ expressing a bright fluorescent genetically-encoded GFP membrane marker. We show these cells form multiple nanotubes in culture, which functionally mediate the propagation of Ca^2+^ signals between cells, and actively traffic GFP-tagged membrane aggregates along their length. Furthermore, using this cell line in a well-established model of brain cancer metastasis in the mouse^15–17^ we show the membrane marker is retained after several weeks *in vivo* and, utilizing high-resolution LLSM of acute live brain slices, we visualize membrane nanotubes interconnecting cells within dense brain metastatic lesions.

## Results and Discussion

### MDA-231 cells form multiple nanotubes in culture

**Figure 1:**
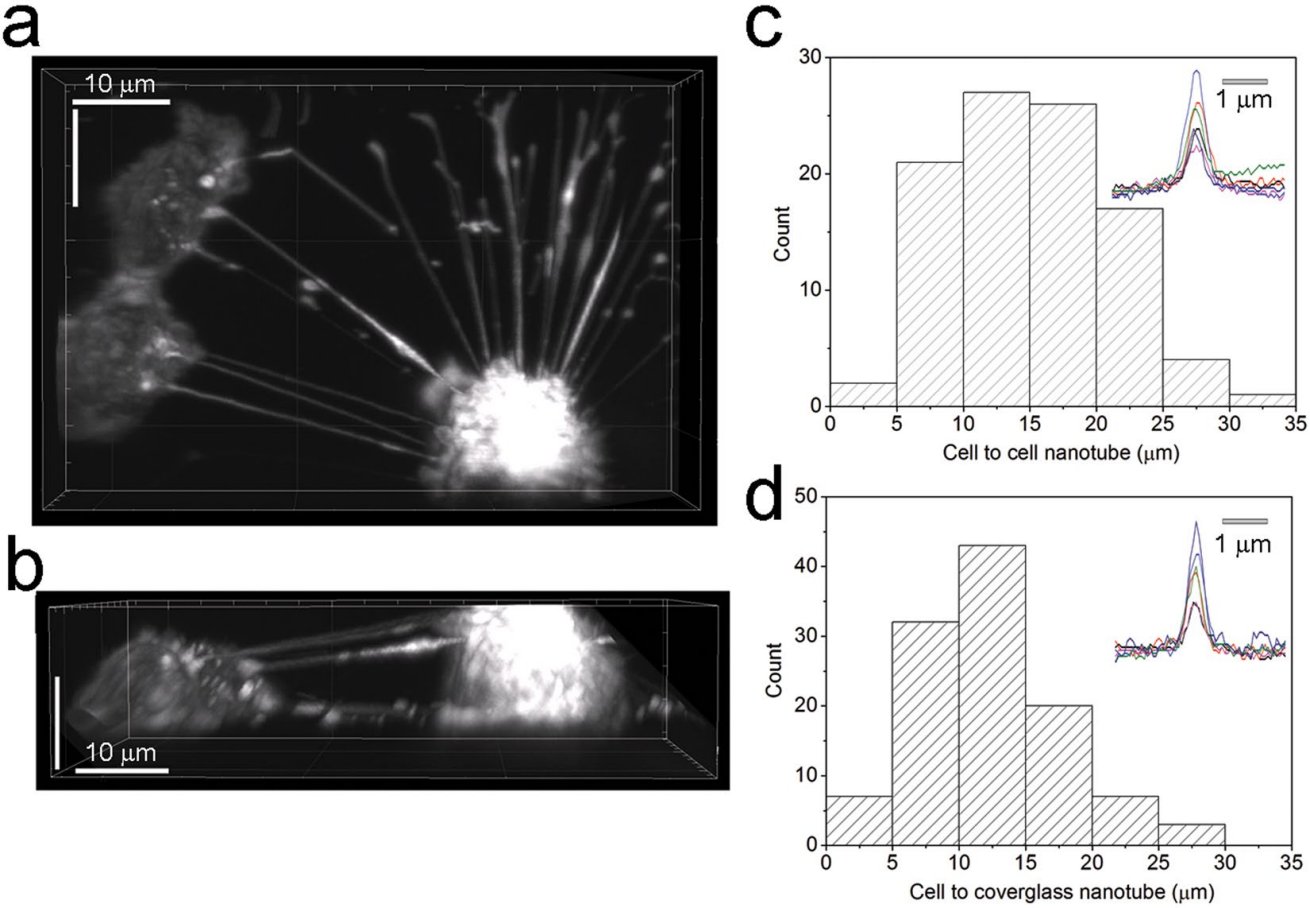
Membrane nanotubes form connections between MDA-231 cells in culture. (**a**,**b**) Representative lattice light-sheet image of MDA-231 cells visualized by a GFP-tagged membrane marker, illustrating nanotubes interconnecting with other MDA-231 cells and contacting the coverglass. See also Movie 1. (**a**) is a maximum intensity z-projection (top view) of the imaging volume, and (**b**) shows an orthogonal (side) view of the same volume. (**c**,**d**) Histograms plot the distributions of lengths of nanotubes that interconnect MDA-231 cells (**c**, n = 98 nanotubes from 16 imaging fields) and those that contact the cover glass (**d**) 113 nanotubes). Insets in (**c**,**d**) are transverse linescan profiles showing the widths of nanotubes that interconnect MDA-231 cells (n = 6, mean FWHM 466 ± 31 nm) and those that contact the coverglass (n = 6, mean FWHM 423 ± 15 nm).

MDA-MB-231Br2 GFP cells (MDA-231GFP) were stably transfected with lentiviral particles driving expression of GFP targeted to the plasma membrane, using the N-terminal palmitoylation sequence of neuromodulin^18, 19^. Cells were plated on glass coverslips and were imaged at room temperature using a custom-built lattice-light sheet microscope. The fluorescent marker brightly highlighted the plasma membrane of the cells and revealed numerous thin extensions. 3D image reconstructions (Fig. 1a and Movie 1) demonstrated numerous projections between MDA-231GFP cells, with side views showing these projections suspended above the substratum; a defining characteristic of membrane nanotube protrusions^3^. The mean cell-to-cell length of membrane nanotubes connecting between pairs of MDA-231GFP cells was 14.85 ± 6.3 µm (mean ± s.d, n = 98 nanotubes from 16 imaging fields, 67% of cells coupled by nanotubes; Fig. 1c). Cross sections of these nanotubes had a width of 466 ± 76 nm (FWHM, mean ± s.d., n = 6; Fig. 1c). We further observed similar membrane protrusions extending from the upper parts of MDA-231GFP cells and forming foot-like contacts to the cover glass (length 12.5 ± 6 µm, mean ± s.d, n = 113; width 423 ± 35 nm FWHM, mean ± s.d, n = 6, Fig. 1b,d). The apparent widths of the nanotubes correspond closely to the lateral point-spread function of the microscope (~460 nm), implying that the true diameter of these structures is appreciably smaller.

### Membrane nanotubes communicate intercellular Ca^2+^ signals between MDA-231 cells and traffic cargo along their length

**Figure 2:**
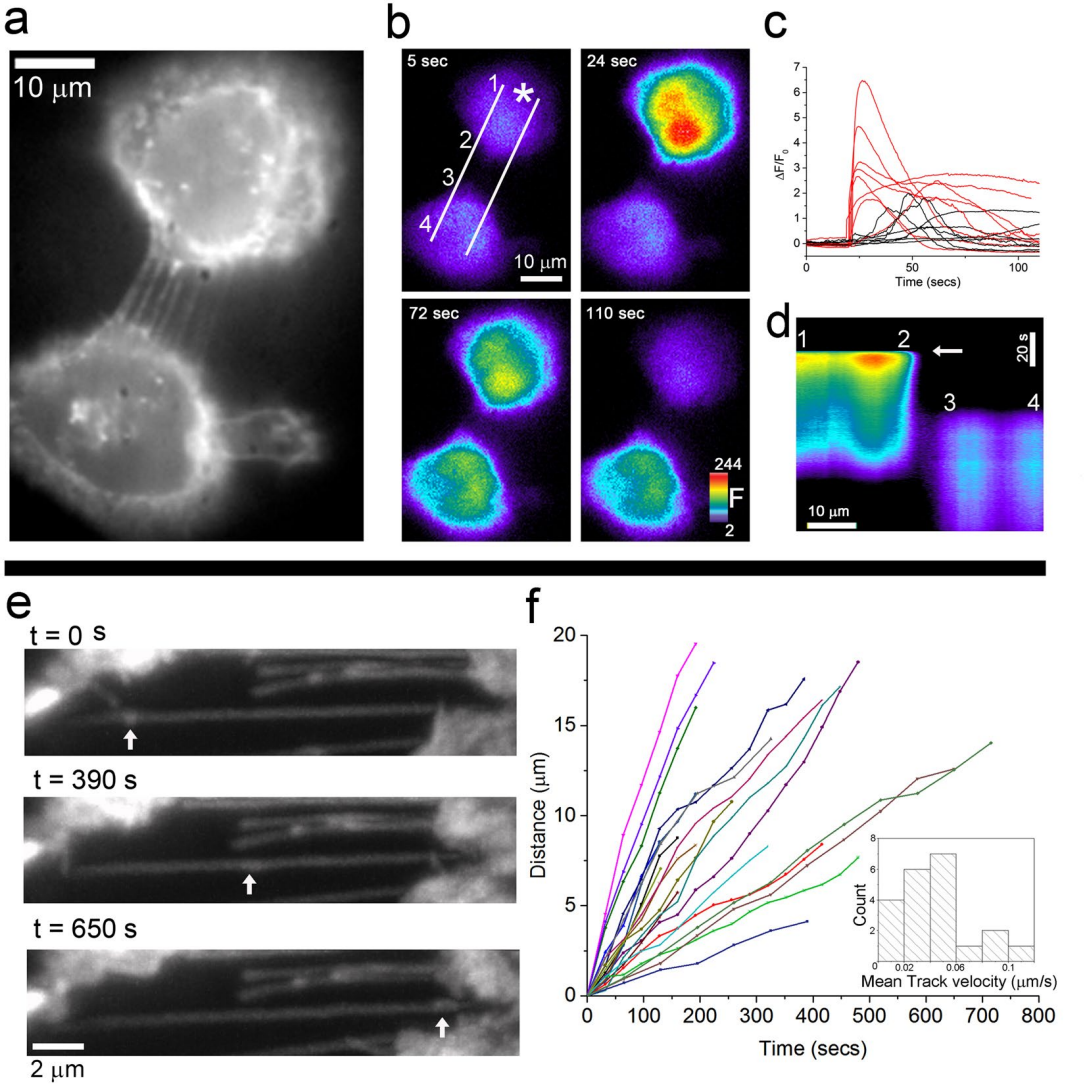
Transmission of Ca^2+^ signals and membrane constituents along membrane nanotubes. (**a**) Nanotubes between two MDA-MB-231PA cells visualized using widefield fluorescence microscopy by a deep red plasma membrane stain. (**b**) The same two cells showing fluorescence of the Ca^2+^ indicator Cal-520 (F, arbitrary units) before and after UV laser spot photorelease of i-IP_3_ delivered at t = 22 s at the location marked by the asterisk in the first panel. White lines and numbers indicate kymograph section and annotations used to generate panel d. (**c**) Traces show Ca^2+^ fluorescence signals evoked from 7 representative cells directly stimulated at 22 s by photorelease of i-IP_3_ (red), and signals in responding, nanotube-connected cells (black). (**d**) Kymograph image (with time depicted vertically and distance horizontally) formed by measuring Ca^2+^ fluorescence changes (ΔF/F_0_) between the lines marked white in (**b**) as a function of time following UV spot stimulation of the upper cell. Notations (1, 2, 3, 4) mark corresponding locations in the cell image and kymograph to delineate sites within each cell and the nanotube connecting them. Arrow marks the time of the UV flash (**e**) Snapshot images captured via lattice light-sheet microscopy illustrating movement of a GFP-tagged membrane constituent along the length of the nanotube at three times as indicated. Arrows mark the locations of the vesicle. Scale bar, 2 µm. See also Movie 2. (**f**) Distance-time plots of membrane constituents moving along nanotubes. Inset graph shows a distribution histogram of mean track velocities. Data are from 22 representative nanotubes in 3 separate cultures.

We investigated whether nanotubes support intercellular communication between cultured MDA-231 cells by looking for transmission of cytosolic Ca^2+^ signals, as demonstrated previously in a HeLa cell model engineered to upregulate nanotube formation^20^. For compatibility with use of a green-emitting Ca^2+^ probe we used parental MDA-MB-231PA cells not expressing the GFP marker, and instead visualized nanotubes using a Deep Red plasma membrane stain. Cells were loaded with the Ca^2+^ indicator Cal-520 and caged IP_3_ (ci-IP_3_), and a focused spot of 405 nm laser light was used to locally uncage i-IP_3_ within individual cells^20^. Robust fluorescence Ca^2+^ signals began almost immediately after the spot flash in all stimulated cells (Fig. 2a–c; ΔF/F_0_ 3.75 ± 0.38, mean ± s.e.m, n = 15). Surrounding cells connected via nanotubes frequently (8/15) showed Ca^2+^ increases (ΔF/F_0_ 1.22 ± 0.23, mean ± s.e.m, n = 8) that began after an appreciable delay (45 ± 15 s, mean ± s.e.m) following the photolysis flash. In contrast, surrounding cells at comparable distances that were not connected to the stimulated cell via nanotubes failed to show detectable Ca^2+^ signals (n = 14), thus excluding paracrine signaling or bleed-over of photolysis light as alternative mechanisms for Ca^2+^ signal transmission.

Ca^2+^ elevations began at sites within the responding cell distant from the sites of nanotube contact (6.18 ± 0.6 µm, mean ± s.e.m), and without any perceptible rise in Ca^2+^ within the nanotubes (Fig. 2d). We thus interpret the Ca^2+^ responses in nanotube-connected cells to arise from transfer of IP_3_ along the nanotube that subsequently evokes Ca^2+^ liberation in the responding cell, rather than from direct transmission of Ca^2+^; analogous to the mechanism we had previously proposed for HeLa M-Sec cells^20^.

In addition to the intercellular transfer of small molecules like IP_3_, several reports describe intercellular trafficking of cargoes via nanotubes^21, 22^. Figure 2e (see also Movie 2) illustrates the trafficking of GFP-tagged membrane aggregates along the length of a membrane nanotube; behavior that was representative of most nanotubes that were clearly visualized. Motion of these constituents was incompatible with a diffusive random walk, but proceeded at a nearly uniform, unidirectional velocity (Figure 2f). The mean track velocity was 2.7 ± 0.3 µm/min (mean ± s.e.m, n = 21), indicative of active motor protein driven transport^23^. We have not yet established whether this transport along nanotubes results in transfer of membrane constituents between cells.

### Imaging of membrane nanotubes within metastatic brain tumors

The MDA-231Br2 breast cancer cell line is widely used to study brain metastasis. Following injection of MDA-231 cells into the left ventricle of an anesthetized mouse these cells cross the blood-brain barrier where they utilize the microvasculature to invade the brain^15–17^. We initially confirmed brain tumor colonization at 3 weeks using bioluminescence from MDA-231 cells stably expressing the luciferase vector^16^. However, whereas bioluminescence imaging is sufficient to image macro scale tumor localization it lacks the resolution to image single cells, let alone membrane nanotubes. Instead, we utilized the stable integration of plasma membrane targeted GFP into the genome of MDA-231 cells as a permanent membrane marker to enable high-resolution LLSM imaging within brain metastases.

**Figure 3:**
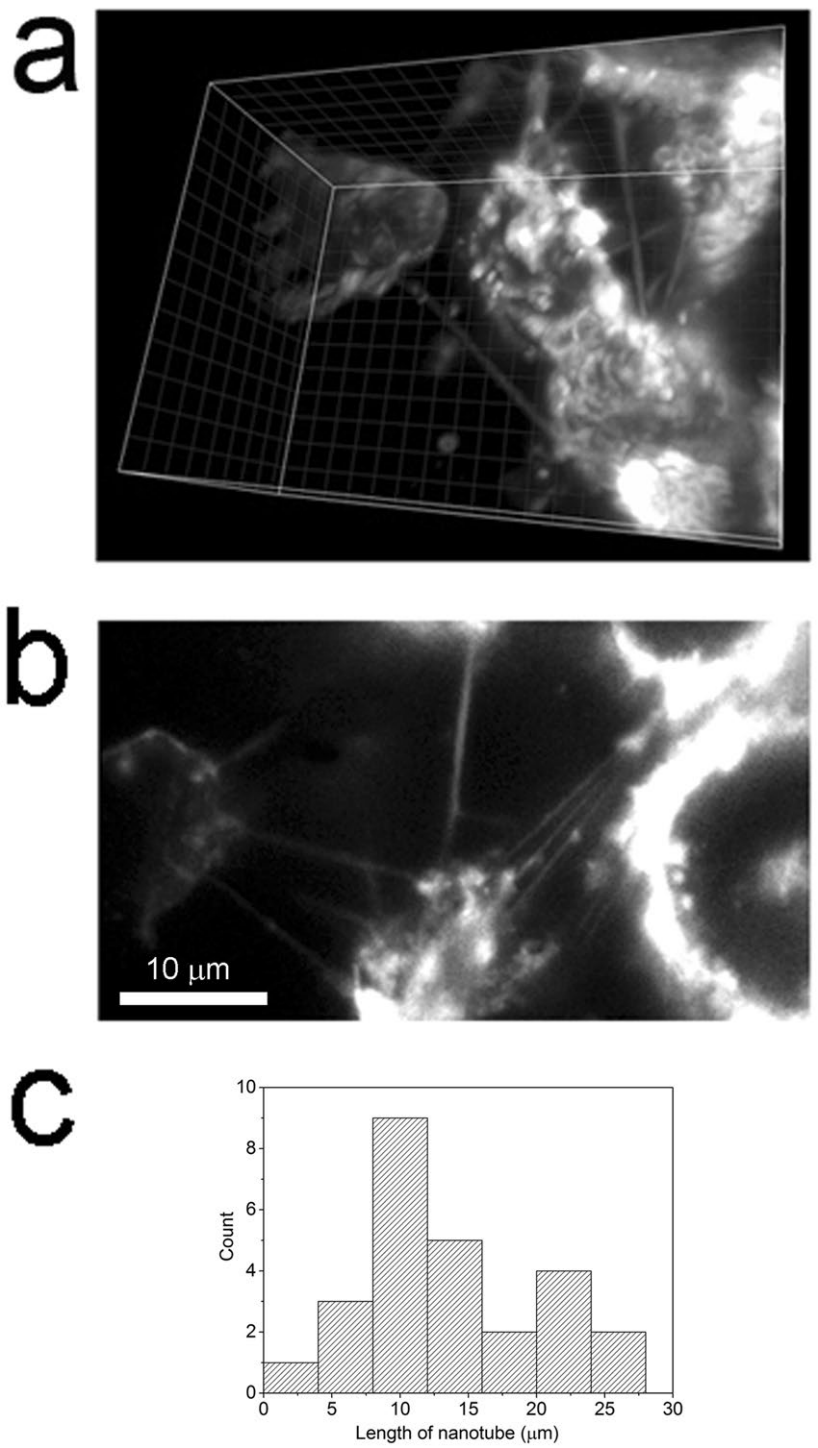
Visualization of membrane nanotubes between MDA-231 cancer cells in a mouse  model of brain metastasis. (**a**) 3D reconstruction of a lattice-light sheet image taken ~10 μm below the cut surface of a live 100 µm thick brain slice at day 24 post-injection. Several nanotubes are visible, connecting between MDA-231GFP cells. Each grid = 2 µm. (**b**) Single, diagonal image plane from the 3 D reconstruction in (**a**) showing multiple nanotubes connecting MDA-231 cells. See also Movie 3 for unprocessed image sweep. (**c**) Histogram plots the distributions of lengths of nanotubes that interconnect MDA-231 cells (**c**), n = 27 nanotubes measured from 4 brain slices from 4 mice).

We prepared brain slices (∼100 µm) from female NU/NU mice that had been injected ∼ 3 weeks previously with 5 × 10^5^ MDA-231GFP cells and rapidly mounted them in medium onto the stage of the LLSM. We observed long (13.85 ± 6 µm, mean ± s.d, n = 26), thin (457 ± 30 nm, mean ± s.d, n = 10) nanotube-like structures interconnecting MDA-231GFP cells within brain slices (Fig. 3a–c and Movie 3); characteristics closely similar to those of nanotubes in culture (cf. Fig. 1).

The MDA-231 cell line has previously been used to document the intercellular exchange of endosomes and micro RNA’s *in vivo*; exchange that, based on cell culture experiments, was proposed to be mediated via membrane nanotubes^9, 24^. Our direct visualization of nanotube-like networks within metastastic tumors, taken together with observations of Ca^2+^ signaling communication and membrane trafficking along membrane nanotubes in culture, suggest that similar signaling networks may exist within brain metastases. The pathophysiological significance of this communication remains to be determined, but findings that analogous tumor microtubes between astrocytoma cells play a role in mediating resistance to standard therapeutic strategies^5, 7^ suggest that the membrane nanotubes we observe in brain metastases may play a similar role. Brain metastasis from breast cancer is a major cause of mortality, with median survival <15 months after diagnosis^25^. A deeper understanding of potential interactions between cancer cells mediated by membrane nanotubes may illuminate new avenues of research and potential therapies.

## Methods

### Cell Culture

MDA-MB-231 Br2 cells were obtained from Memorial Sloan Kettering Cancer Center (New York, USA), and MDA-MB 231 PA cells from ATCC (Virginia, USA). Cells were maintained at 37 °C in humidified environment with 95% air and 5% CO_2_ in DMEM (Thermofisher; #21063) supplemented with 10% FBS (Omega Scientific, # FBS-11(lot#593515)). All *in vitro* imaging was conducted on live cells that were harvested from tissue culture flasks by incubation with 0.25% Trypsin-EDTA (Gibco; #25200–056) and sub-cultured (200,000 cells per dish) on 35 mm glass-bottom imaging dishes (MatTek; #P35-1.5-14-C), or on 10 mm glass cover slips 24 hours prior to imaging.

### Material and reagents

The acetoxymethyl (AM) ester form of the organic Ca^2+^-dye Cal-520 (AAT Bioquest #21130) was reconstituted with dimethyl sulfoxide (DMSO) containing 20% pluronic F-127 (DMSO/F-127; Invitrogen; #P-3000MP) to a final concentration of 1 mM and stored, shielded from light, at −20 °C. The membrane-permeant caged IP_3_ analogue ci-IP_3_/PM (D-2,3-O-Isopropylidene-6-O-[2-nitro-4,5 dimethoxy] benzyl-myo-Inositol 1,4,5-trisphosphate Hexakis [propionoxymethyl] ester) was purchased from siChem (#cag-iso-2-145-10), solubilized with DMSO/F-127 to a final concentration of 400 µM and stored at −20 °C. Deep Red plasma membrane stain was purchased from Invitrogen (#C10046) and stored at −20 °C. Lentiviral particles driving expression of GFP to the plasma membrane (rLV.EF1.AcGFP1-Mem-9) were obtained from Takara Bio USA. All other reagents were purchased from Sigma-Aldrich.

### Ca^2+^ Imaging

MDA-MB-231 PA cells were loaded by incubation with 1 µM Cal-520/AM and 1 µM ci-IP_3_/PM for 1 hr at 37 °C. Following loading cells were washed with pre-warmed culture medium and then incubated with Deep Red plasma membrane stain, diluted (1:10,000) in pre-warmed culture medium, for 10 minutes. Cells were then rinsed three times with a Ca^2+^-containing HEPES buffered salt solution composed of (mM): 135 NaCl, 5.4 KCl, 2 CaCl_2_, 1 MgCl_2_, 10 HEPES, and 10 glucose (pH = 7.4 set with NaOH at room temperature) and immediately used for imaging in this medium.

Imaging of cytosolic Ca^2+^ signals was accomplished using a custom-built microscope system based around an Olympus IX 50 microscope equipped with an Olympus 100X objective (NA 1.40) as described previously^26, 27^. Excitation light from the expanded beam of either 488 nm (Coherent, Santa Clara, CA) or 561 nm (Opto-Engine, Midvale, UT) diode pumped solid-state (DPSS) lasers was introduced by a small reflective prism and brought to a focus at the rear focal plane of the objective. Emitted fluorescence was collected through the same objective and filtered by steep-cut long pass filters (Semrock, Rochster, NY) with cut-off wavelengths of 488 nm or 568 nm corresponding to the respective laser wavelengths. Images were acquired by an Evolve 512 electron-multiplied c.c.d. camera (Photometrics, Tucson, AZ) with 512 × 512 pixel resolution (1 pixel = 0.166 µm) at a rate of 10 frames per sec (fps). Image data were streamed to computer memory using MetaMorph software (Universal Imaging/Molecular Devices, Sunnyvale, CA) and were subsequently stored on hard disc for offline analysis.

Spot UV photolysis of ci-IP_3_ was achieved using a 405 nm laser (CivilLaser, Hangzhou, China) and galvanometer-driven mirrors interfaced with a custom-written algorithm to control the position and duration of the laser spot in order to selectively stimulate a single cell without the spurious activation of any surrounding cells.

### Ca^2+^ Imaging analysis

Image data in MetaMorph stk format were processed using a custom algorithm, written in the Python programming language, for the detection and analysis of whole-cell Ca^2+^ signals from fluorescence video recordings^28, 29^. Image stacks were processed by first subtracting the background fluorescence from the entire image stack and then normalized by dividing the stack by the mean fluorescence of each pixel averaged over 100 frames prior to stimulation (F_0_). Individual cells were manually identified and a region of interest (ROI), outlining the entire cell, was generated for all cells in the imaging field. Normalized fluorescence values from ROIs, corresponding to individual cells, was used to calculate amplitude and lag time of the Ca^2+^ response. Peak amplitude was measured as the change in fluorescence from baseline immediately preceding the event to the maximum fluorescence value of the Ca^2+^ response. Lag time was quantified as the duration of time between the peak amplitude of the Ca^2+^ response in the stimulated cell to the peak response in the connected cell. Linescan kymograph images were derived by measuring fluorescence across an area to encompass all nanotube connecting cells (width 15 pixels, 2.4 μm) from ΔF/F_0_ image stacks (created as described above), displaying their evolution over time as a pseudocolored representation utilizing the kymograph function in MetaMorph. All data presented as mean ± sem, and statistical analysis was performed using the paired Student’s t-test; P < 0.05 was considered significant.

### Lattice light-sheet microscopy

The custom LLS microscope was constructed largely following the original HHMI prototype design^14^, but with simplifications including the use of a custom graticule to generate lattice patterns in place of a spatial light modulator, and use of standard 40× NA 0.8 water-dipping objectives (Nikon) for both projection and imaging lenses. In brief, the beam from a 478 nm laser was expanded and then passed through two pairs of cylindrical lenses to create a parallel beam of light as a thin slit impinging on the custom graticule to generate the lattice pattern. The resulting diffraction pattern (Fourier transform of the lattice) was spatially filtered by an annular aperture and re-imaged at the back focal plane of the projection objective. Fluorescence excited in the specimen by the resulting light-sheet (width ~1.2 μm, FWHM) was imaged through the orthogonal objective and relayed to a sCMOS camera (Andor Xyla) via a tube lens providing a final magnification of 105 nm per pixel. Cells were cultured on a coverglass, which was mounted on a cantilever driven by a piezoelectric translation stage. Custom software (available at http://flika-org.github.io/index.html) was used to synchronize camera acquisition with stepping of the piezo stage, and to reconstruct orthogonal x,y,z 3D images from the series of 45° diagonal image ‘slices’ captured by physically scanning the specimen through the light sheet. Imaging was performed at room temperature, with cells immersed in culture medium.

Tracking of visually-identified GFP-tagged membrane constituents was performed manually using Imaris software (Bitplane) version 7.2.

### Animal Studies

All experiments were performed according to a protocol approved by the University of California Irvine Institutional Animal Care and Use Committee. Athymic female nude mice (Jackson Laboratory) were used at 4–6 weeks of age.

Brain metastatic assays were conducted as previously described^15–17^. For 24 day brain metastasis studies 2.5 to 5 × 10^5^ MDA-231Br2 cells stably expressing plasma membrane targeted GFP in 100 µL ice cold PBS were injected into the left cardiac ventricle of anesthetized (Avertin 350 mg/Kg) mice. Metastases were allowed to develop for ∼3 weeks with tumor colonization monitored using the triple-fusion reporter construct encoding herpes simplex virus thymidine kinase 1, GFP and firefly luciferase (TGL vector) stably expressed by this cell line; injection of luciferin to induce bioluminescence from luciferase (150 mg/kg) was imaged using an *In Vivo* Imaging system (IVIS, PerkinElmer, MA, USA).

For imaging of acutely dissected brain slices, mice were euthanized with an overdose of avertin (600 mg/kg). Brains were quickly removed and placed into ice-cold artificial cerebrospinal fluid (ACSF; 119 mM NaCl, 26.2 mM NaHCO_3_, 2.5 mM KCl, 1 mM NaH_2_PO_4_, 1.3 mM MgCl_2_, 10 mM glucose) bubbled with carbogen. Coronal cortical slices (~100 µm) were cut using a vibroslice (Camden Instruments).

## Electronic supplementary material

Supplementary Information

Supplementary Movie 1

Supplementary Movie 2

Supplementary Movie 3
