# Supplementary material for: Lattice light sheet imaging of membrane nanotubes between human breast cancer cells in culture and in brain metastases
Source: Sci Rep. 2017 Sep 8;7:11029. doi: 10.1038/s41598-017-11223-y (PMC5591308; doi:10.1038/s41598-017-11223-y)
Supplement: Supplementary file 1 — Supplementary Information [file 41598_2017_11223_MOESM1_ESM.doc]

**Lattice light sheet imaging of membrane nanotubes between human breast cancer cells in culture and in brain metastases.**

**Authors:** Ian Parker^1,2^, Katrina T. Evans^1^, Kyle Ellefson^2^, Devon A. Lawson^1^, and Ian F. Smith^2^

**Author Affiliation:**

Ian Parker
^1^Department of Neurobiology and Behavior, ^2^Department of Physiology and Biophysics. University of California,

Irvine, CA
[iparker@uci.edu](mailto:iparker@uci.edu)

Katrina T. Evans
^2^Department of Physiology and Biophysics
University of California,

Irvine, CA
[krtaylor@uci.edu](mailto:krtaylor@uci.edu)

Kyle Ellefson

^1^Department of Neurobiology and Behavior
University of California,

Irvine, CA
kellefse@uci.edu

Devon A. Lawson

^2^Department of Physiology and Biophysics
University of California,

Irvine, CA

[dalawson@uci.edu](mailto:dalawson@uci.edu)

Ian F. Smith
^1^Department of Neurobiology and Behavior
University of California,

Irvine, CA
[ismith@uci.edu](mailto:ismith@uci.edu)

**Corresponding author**:

Ian F. Smith, [ismith@uci.edu](mailto:ismith@uci.edu)

**Supplementary material.**

Movie 1. Lattice light sheet movie of live MDA231GFP cells expressing plasma membrane tagged GFP. Membrane nanotubes form between MDA231 cells and also project onto the cover glass.

Movie 2. Lattice-light sheet movie of membrane-bound constituent trafficking along the length of membrane nanotubes in MDA 231 cells expressing plasma membrane tagged GFP.

Movie 3. Diagonal lattice-light sheet sweep through a brain tumor in a live 100 µm thick brain slice at day 24 post-injection revealing nanotubes interconnecting MDA 231 cells
